# The Level of Mesenchymal-Epithelial Transition Autophosphorylation is Correlated with Esophageal Squamous Cell Carcinoma Migration

**DOI:** 10.52547/ibj.25.4.243

**Published:** 2021-06-21

**Authors:** Negin Taghehchian, Meysam Moghbeli, Baratali Mashkani, Mohammad Reza Abbaszadegan

**Affiliations:** 1 Department of Chemistry, Faculty of Science, Ferdowsi University of Mashhad, Mashhad, Iran;; 2 Department of Medical Genetics and Molecular Medicine, School of Medicine, Mashhad University of Medical Sciences, Mashhad, Iran;; 3 Department of Clinical Biochemistry, Faculty of Medicine, Mashhad University of Medical Sciences, Mashhad, Iran;; 4 Medical Genetics Research Center, Mashhad University of Medical Sciences, Mashhad, Iran

**Keywords:** c-MET, Esophageal squamous cell carcinoma, Receptor tyrosine kinases

## Abstract

**Background:**

The MET receptor is a critical member of cancer-associated RTKs and plays an important role in different biological activities, including differentiation, migration, and cell proliferation.

**Methods:**

In this study, novel MET inhibitors were introduced and applied on esophageal squamous carcinoma cell line KYSE-30, and the level of proliferation and migration, as well as the activated form of MET receptor protein were assessed in the examined cells. The human KYSE-30 cell line was cultured according to ATCC recommendations. The mRNA level of the MET gene was measured in the examined cell line using the quantitative RT-PCR assay. Cytotoxicity evaluation test was performed at different concentrations of heterocyclic anti-MET compounds (i.e. D1, D2, D5, D6, D7, and D8). Finally, the capability of these compounds in MET receptor inhibition was evaluated using the migration assay and Western blot. All experiments were performed in triplicate and repeated three times with similar results.

**Results:**

Cell growth and proliferation were significantly inhibited (*p* ≤ 0.05) by all the above-mentioned compounds. Moreover, the majority of compounds significantly prevented the cell migration (*p* ≤ 0.05) and inhibited MET autophosphorylation. Interestingly, the level of phosphorylated MET was significantly correlated with KYSE-30 cell migration.

**Conclusion:**

The obtained data introduced and confirmed the biological activities of the mentioned novel compounds in KYSE-30 cells and proposed that the therapeutic inhibition of MET with these compounds may be a powerful approach for inhibiting cancer cell migration and proliferation although some structural optimizations are needed to improve their inhibitory functions.

## INTRODUCTION

ESCC is the sixth leading cause of cancer deaths globally^[^^1^^]^. Despite recent advances in medical and surgical treatments, including new surgical methods, preoperative chemo-irradiation, and postoperative chemotherapy, the patients’ five-year survival rate remains low (15-25%) due to the poor prognosis of the disease^[^^2^^]^. Therefore, specific prognostic and diagnostic biomarkers are required for either the targeted therapy of the disease or improvement of the patients’ survival. 

RTKs serve as essential transmembrane receptors and rely on signals from the extracellular environment into the cells. Their inhibition may cause the deregulation of cell signaling pathways and lead to metabolic defects^[^^3^^,^^4^^]^, developmental abnormalities^[^^5^^]^, and tumorigenesis^[^^6^^]^. MET protein was first discovered in a human osteogenic sarcoma cell line as a part of a hybrid gene generated by the fusion of translocated promoter region from chromosome 1q25 and MET ^[^^7^^,^^8^^]^. Cells with the epithelial origin normally express MET^[^^9^^] ^although endothelial cells^[^^10^^]^, adipose tissues^[^^11^^]^, hepatocytes, hematopoietic cells^[^^12^^]^, melanocytes, and neonatal cardiomyocytes express MET receptors, as well^[^^13^^]^. MET receptor activation occurs through the binding of its cognate ligand to the extracellular domain, triggering kinase activity, and phosphorylation of two tyrosine residues (i.e. Y1234 and Y1235) in the cytoplasmic domain of the receptor^[^^14^^,^^15^^]^. Docking sites for intracellular adaptor proteins are phosphorylated residues that can modulate the cell survival, differentiation, and proliferation through the activation of downstream components involved in different cell signaling cascades, including the MAPK, PI3K-AKT, and STAT signaling pathways^[^^16^^-^^19^^]^. The irregular activation of MET kinase happens by a variety of mechanisms such as the ligand-mediated HGF, autocrine or paracrine stimulation, gene mutation, and amplification, as well as cross-talk with other receptors^[^^20^^]^. The malfunction of MET kinase has been reported in several malignancies, comprising thyroid, renal cell, colorectal, ovary, breast, pancreas, prostate, gastric, and liver cancers, along with ESCC^[^^16^^]^. 

Pathological MET activation has been suggested as a therapeutic target for cancer treatment^[^^21^^]^ since it promotes proliferation, invasion, migration, and tumor cell metastasis^[^^22^^]^. Current clinical drugs against the HGF/MET pathway include SMIs and monoclonal antibodies^[^^21^^,^^23^^]^. The SMIs can block MET kinases through binding to its ATP-binding pocket and can suppress the MET-dependent biological activities of the cell^[^^24^^]^. SMIs are considered as one of the most emerging molecular therapeutic strategies against cancer cells, and extensive efforts have focused on designing, synthesis, and biological evaluation of these molecules against specific kinases^[^^25^^]^. The present study was designed to evaluate the impact of MET phosphorylation on the biological behavior of the ESCC cell line KYSE-30 through introducing and investigating the efficacy of some novel heterocyclic inhibitor molecules against MET kinase activity.

## MATERIALS AND METHODS

**Compounds**

In this study, we used six novel heterocyclic compounds, which were designed and synthesized by Saadatmandzadeh and co-workers, the Department of Chemistry, Ferdowsi University, Mashhad, Iran. The structural features of compounds are summarized in Table 1, and the development procedure of heterocyclic compounds was described before^[^^26^^]^. Drug-likeliness was examined by applying Lipinski’s rule of five for all compounds^[^^27^^]^. DMSO was used to make a stock solution of the compounds because of better solubility^[^^28^^,^^29^^]^, and then DMSO toxicity was assessed by MTT assay. In this study, eight compounds (i.e. D1, D2, D3, D4, D5, D6, D7, and D8) were selected for experiments. The nuclear magnetic resonance and mass spectrometry were employed to confirm the chemical structure of compounds^[^^26^^]^.

**Cell culture**

For experiments, human esophageal squamous carcinoma cell line KYSE-30 was chosen due to its invasive nature and obtained from the National Cell Bank, Pasteur Institute of Iran (Tehran). The cells were cultured in the RPMI (Roswell Park Memorial Institute, USA)-1640 medium (PAA, Austria) supplemented with 10% fetal bovine serum (Invitrogen, CA) and 100 U/mL-100 mg/mL of penicillin-streptomycin (PAA) in a humidified atmosphere with 5% CO_2_ at 37 °C^[^^30^^]^.

**RNA extraction, cDNA synthesis, and RT-PCR**

The total RNA was extracted from the KYSE-30 cell line using the total RNA extraction kit (Pars Tous, Iran). Approximately 2 × 10^6^ cells were lysed by adding 750 μL of the lysis buffer and 150 μL of chloroform and then centrifuged at full speed for 12 minutes. All steps were performed according to the manufacturer’s instructions. The purity and quantity of total RNA were measured by a NanoDrop spectrophotometer (WPA, Biowave II^+^, Germany). Moreover, the integrity of the RNA was evaluated by the electrophoresis on 1% agarose gel, and 28S and 18S ribosomal RNA bands were observed accordingly. After the treatment of the extracted RNA by *deoxyribonuclease* I, cDNA was synthesized using the Thermo Fisher Scientific (USA) kit based on the manufacturer’s protocol. Next, cDNA was subjected to PCR amplification in a 20-µL final reaction volume, including 1 µL of cDNA, 0.5 µL of each primer (20 mM), 6 µL of dH_2_O, and 12 µL of Taq DNA Polymerase Master Mix RED (Ampliqon, USA). The glyceraldehydes 3-phosphate dehydrogenase gene was used as the internal control, and the target gene (MET) evaluated by using a speciﬁc primer set, including sense 5’TTGGATAGGCTTGTAAGTGCCC3’ and antisense 5’TACTGCACTTGTCGGCATGAA3’. Amplifications were conducted in 45 cycles as the initial denaturation step at 95 °C for 10 minutes, the denaturation step at 94 °C for 15 seconds, the annealing step at 60 °C for 30 seconds, extension at 72 °C for 30 seconds, and the final extension at 72 °C for 5 minutes. Eventually, PCR products were detected on 2% agarose gel electrophoresis.

**Table 1:** The chemical structure of compounds (D1, D2, D5, D6, D7, and D8)

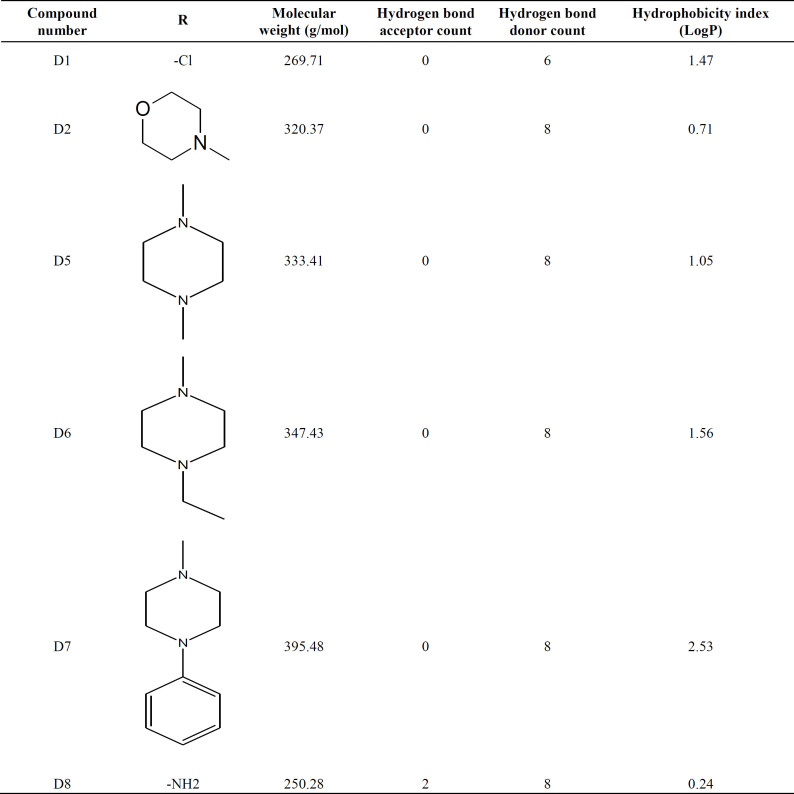

IUPAC, International Union of Pure and Applied Chemistry

**Cell proliferation assay**

Nearly 5 × 10^4^ KYSE-30 cells per well were applied and treated with the increasing concentration (ranging from 1.95 to 16000 µM/µL) of the compounds in 96-well plates for 48 hours at 37 °C^[^^31^^]^. The cell viability was assessed by Resazurin reagent (Sigma-Aldrich). The fluorescent intensity of resorufin, as the reduced form of resazurin, was measured in viable cells by a plate reader (Perkin Elmer Victor X5, USA). In addition, the IC_50_ value for each compound was calculated by Graph Pad Prism (Graph Pad Software, CA). The experiment was performed in triplicates, and the mean of three independent assays was measured as the final IC_50_ value.

***In vitro*** **wound healing assay**

The effects of heterocyclic compounds on the migration of KYSE-30 cells were examined using the scratch assay. KYSE-30 cells were seeded onto six-well plates at a density of 1 × 10^6^ and incubated at 37 °C for 24 hours. Next, to create a cell-free zone, confluent cells were scraped with a sterile 100-μL pipette tip and after scraping, the cellular debris was removed by washing three times with PBS. Cells were then incubated with the IC_50_ concentrations of the compounds for 24 hours. The process of cell migration was followed by using an inverted microscope and the images were obtained immediately after scraping (0 hour) and at different time points (e.g. 24 and 72 hours). The experiments were performed in triplicate. The area of the scratches in different images was measured using Image J software (version 1.48, the National Institutes of Health, Bethesda, MD, USA). To quantify the migration ability of the cultured cells, the cell-free areas of the scratches at different times were calculated as a percentage of the initial scratched area at 0 hour in each group of drugs. The experimental data were plotted to describe the percentage of the cell-free area versus time points^[^^32^^]^.

**Western blot**

KYSE-30 cells were seeded onto six-well plates at a density of 1 × 10^6^ cells/well and allowed to attach overnight. The cells were treated with the IC_50_ concentrations of the compounds as indicated above. The cultured cells were washed with PBS, lysed with a 70-µL ice-cold radio-immunoprecipitation assay buffer (RIPA lysis buffer; 50 mM of Tris HCl, 150 mM of NaCl, 1% Triton X-100, and 0.5% sodium deoxycholate, and 0.1% SDS, ethylenediamine-tetraacetic acid, pH 7.4) and treated with a protease inhibitor (Sigma-Aldrich). Then cell lysates were centrifuged at 13,000 × rpm at 4 °C for 10 minutes, and the supernatants were analyzed for the presence of target protein. For immunoblotting analysis, identical amounts of extracted proteins from each sample were subjected to electrophoresis on 10% SDS poly-acrylamide *gel* electrophoresis, and the proteins were transferred to polyvinylidene ﬂuoride membrane. Next, the blot was incubated in the blocking buffer containing 2% bovine serum albumin at room temperature for 2 hours. Then polyvinylidene ﬂuoride membranes were probed overnight with 1:500 dilution of primary monoclonal rabbit antibody against phospho-Y1234/1235 MET (Cell Signaling Technology, USA # 3077) at 4 °C, followed by incubation with anti-rabbit *immunoglobulin* G peroxidase (A0545, Sigma-Aldrich) at room temperature for 1 hour. Using sc-47778 (Santa Cruz Biotechnology, Dallas, TX, USA), blotting was performed for β-actin as a control for protein loading. Finally, the rabbit peroxidase-conjugated secondary antibody was applied, and antibody-bounded proteins were visualized using the enhanced chemiluminescence Western blot analysis system (PerkinElmer Life and Analytical Sciences, Boston, USA). Twenty-five microgram of protein samples were separated on a 10% SDS acrylamide gel (Bio-Rad, USA) at 150 V for 1 hour, and the proteins were transferred to a nitrocellulose membrane (Whatman, UK). After blocking in 5% fat-free milk, the membrane was treated with the diluted primary antibody (1:800) at 4 °C overnight and then with the diluted secondary antibody (1:3000) at room temperature for 1 hour. Subsequently, immunoblot membranes were visualized by an enhanced chemiluminescence reaction using the detection kit (Pars Tous Biotechnology, Iran). The Western blotting assay was repeated at least three times on every sample with similar results. Finally, the relative intensity of immunoreactive protein bands was quantified by Image J software^[^^33^^]^.

**Statistical analysis**

The statistical analysis was conducted using SPSS software (version 20.0, SPSS Inc, Chicago, IL, USA) and GraphPad Prism (version 8.0, GraphPad Software, San Diego, CA). Data were presented as the mean ± SD from three independent experiments. The scratch assay data were statistically compared using the one-way ANOVA test to find differences in the migration of KYSE-30 cells between different treatment groups. A *p* ≤ 0.05 was regarded as statistically signiﬁcant. 

## RESULTS

**Important structural features of novel compounds**

Lipinski’s rule of five was applied on all the compounds, as reported in Table 2. The molecular weight of the compounds was less than 500 kDa, and LogP values for all substances were less than 5, while those of compounds D2 and D8 were less than one. The number of hydrogen bond donors in all compounds was in the appropriate range (i.e. 8-6). The compounds had no hydrogen bond acceptor except for D8, which had two hydrogen bond acceptors. In this study, eight compounds were selected although six of which (D1, D2, D5, D6, D7, and D8) were finally nominated for further experiments.

**Table 2:** The hallmarks of D group compounds and their correlation with Lipinski’s rules

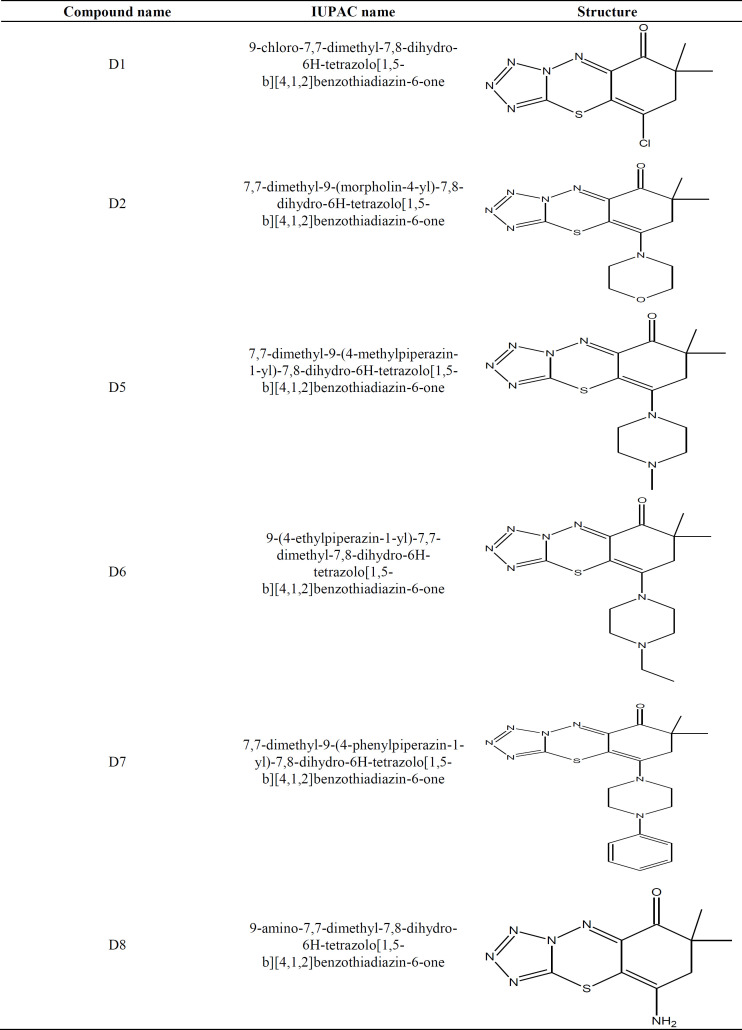

**Table 3:** Enzymatic and cellular results for compounds (D1, D2, D5, D6, D7, and D8)

**Compound**	**IC50** **(µM**)	***p*** ** value** **cytotoxicity assay**	***p*** ** value** **scratch assay**	**MET phosphorylation** **using Western blot**
D8	75	0.05	0.13	Unchanged
D7	162	0.017	0.02	Decreased
D1	167	0.04	0.03	Decreased
D6	307	0.02	0.03	Decreased
D2	556	0.03	0.02	Decreased
D5	674	0.006	0.05	Decreased

**Cell proliferation**

**Fig. 1:**
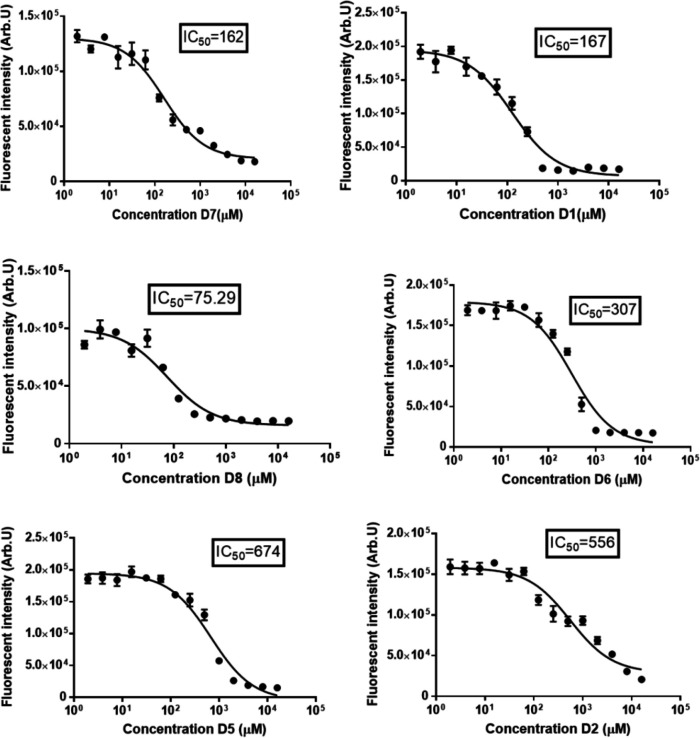
Effect of SMIs (D1, D2, D5, D6, D7, and D8) as a potent, highly selective and ATP-competitive inhibitor of MET on the proliferation of KYSE-30 Cells. Western cells were cultured in the presence of the indicated concentrations of drugs (IC_50_ value). After 48 hours, the relative number of viable cells per well was determined based on the fluorescence of resorufin produced by the reduction of resazurin. Data points are shown as the mean ± SD of four replicate wells. The Figure shows a representative of three independent experiments

The expression of MET mRNA was confirmed in the KYSE-30 cell line by RT-PCR. The cytotoxic effects of all synthesized compounds, including D1, D2, D5, D6, D7, and D8, were assessed on KYSE-30 cell proliferation. The results were expressed as IC_50_ values in Table 3. All compounds significantly decreased the proliferation of compound-treated KYSE-30 cells compared to untreated cells (*p* ≤ 0.05). IC_50_ values for different compounds were determined as 75 µM (D8), 162 µM (D7), 167 µM (D1), 307 µM (D6), 556 µM (D2), and 674 µM (D6). In addition, similar results were found regarding the proliferative potential related to three independent experiments after treatment, and all results were expressed as the mean ± SD of three replicate wells. The inhibitory effect of compounds on MET kinase activity is shown as curves in Figure 1. Based on cytotoxicity assessments, D8 showed the lowest IC_50_ value among all compounds. The IC_50_ concentration had a rising trend in D7, D1, D6, D2, and D5. Eventually, the cell growth and proliferation were significantly inhibited by all these compounds (*p* ≤ 0.05), the details of which are provided in Table 2.

**Fig. 2:**
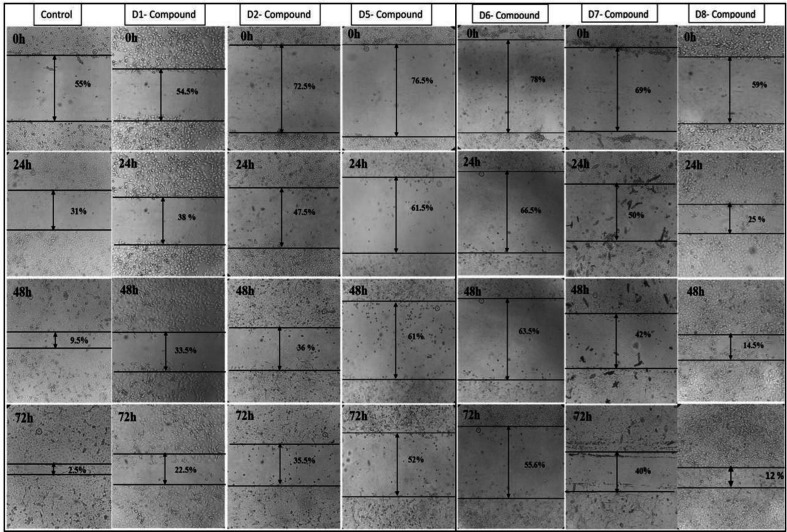
Wound healing assays performed on KYSE-30 cells in the absence or the presence of SMIs (D1, D2, D5, D6, D7, and D8). Compounds D6, D7, and D8 reduced the cellular motility of the KYSE-30 cell line. A scratch wound healing assay was conducted, and inverted microscope images (×10) for 0 hour, and 24, 48, and 72 hours of compound treatment (at the IC_50_ concentration) are given. Cells treated with these compounds were unable to close the wound in the conﬂuent culture when compared with untreated control cells after 72 hours

**Wound healing assay**

**Fig. 3:**
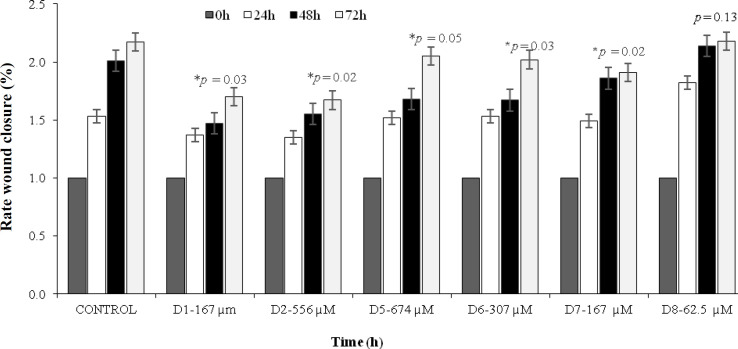
Mobility rate histograms of each SMI and the control. The graphies present the frequency of wound recovery in KYSE-30 cells. Control cells closed the wound faster than the inhibited cells. All results are representative of three independent experiments (^*^p ≤ 0.05).

The wound healing assay was performed to analyze the effect of the applied compounds on the migration of the KYSE-30 cell line. All the compounds (except for D8) significantly decreased the KYSE-30 cell migration (Fig. 2). Among the compounds, D6 and D5 had the highest suppressive role in the cell migration with wound closure percentages of 55.6% and 52%, respectively. Although D8 had no significant impact on KYSE-30 cell motility, it reduced the cell migration by almost fivefolds in comparison with control cells (12% vs. 2.5%, wound closure percentage). As mentioned above, D5 and D6 represented the highest suppressive role compared to other compounds. It seems that D7 had the highest efficiency (regarding Western blot results) for treatment because of its lower IC_50_ value compared with D5 and D6 (167 vs. 674/307 µM IC_50_ values, respectively), the related data of which are illustrated in Figure 3. 

**Effects of the compounds on MET auto-phosphorylation**

**Fig. 4:**
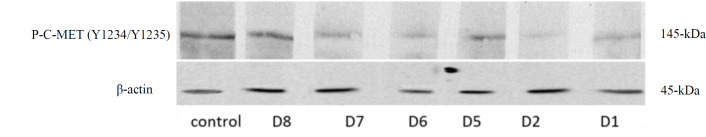
Inhibition of MET autophosphorylation by SMIs. The Western blot analysis of untreated KYSE-30 cells and exposed with the IC_50_ concentration of D group compounds for 24 hours. Compounds could suppress MET phosphorylation compared with the control cells, except for the D8 compound

Having analyzed the protein expression pattern using the Western blot with a specific antibody against phosphorylated MET, small molecule TKIs inhibited the tyrosine kinase autophosphorylation (Tyr1234/1235 residues) of MET and decreased the cell proliferation and survival. IC_50_ values for compounds, corresponding to the MET auto-phosphorylation, are summarized in Table 3. Five out of six novel applied MET SMIs, including D1, D2, D5, D6, and D7 (except for D8), efﬁciently inhibited MET autophosphorylation at tyrosines 1234/1235 in the KYSE-30 cell line. However, D8 demonstrated no significant MET inhibitory effect on KYSE-30 cells (Fig. 4). 

**Correlation between MET autophosphorylation and KYS-30 cell migration**

**Fig. 5:**
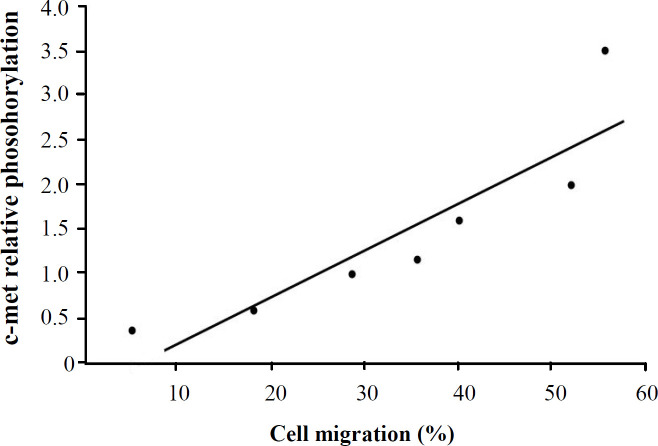
Correlation between MET autophosphorylation and KYSE-30 cell migration. The graph represents a significantly reduced cell migration ability of examined cells following MET inhibition (^*^p ≤ 0.05).

Based on the combined results of Western blot analysis (which detected the phosphorylated form of MET protein) and wound healing assay (which measured the capability of cell migration at the IC_50_ values of the applied compounds), a linkage was found between the level of MET phosphorylation and cell migration. It was revealed that ESCC cell migration was significantly inhibited by these SMIs in the examined cell line. The inhibition of MET RTK phosphorylation reduced the cell migration ability of KYSE-30 cells. A low level of MET phosphorylation, confirmed by the Western blot, decreased the migration potential of KYSE-30 cells though a high level of MET phosphorylation (a strong band in the related Western blot test) was significantly correlated with a large distance of KYSE-30 cell migration (*p* = 0.006). The obtained data are depicted in Figure 5.

## DISCUSSION

ESCC is typically diagnosed when tumor cells are in advanced stages. Invasion and metastasis are important indices of poor prognosis in ESCC patients. Therefore, understanding the involved molecular mechanisms in the tumorigenesis and aggressiveness of the disease may help develop new effective and well-tolerated therapies for treating ESCC^[^^34^^]^. 

In this study, the inhibitory effect of MET was evaluated using novel compounds on the auto-phosphorylation and growth of the ESCC cell line. MET inhibition by these compounds not only resulted in a notably decreased MET autophosphorylation but also significantly inhibited cell migration. It has been shown that the treatment of KYSE-30 cells with our designed MET inhibitors reduced MET auto-phosphorylation on Tyr1234/1235 residues, as well as cell proliferation and migration. Furthermore, the level of MET phosphorylation directly affected the migration ability of KYE30 cells. Indeed, the high level of MET autophosphorylation caused an increased distance of cell migration. 

RTKs play a crucial role in different cellular processes, such as cell metabolism, diﬀerentiation, proliferation, and survival. 58 RTKs have been identified for humans and are classiﬁed into 20 subfamilies. The same molecular architecture is observed in all RTKs consisting of an extracellular ligand-binding domain and a cytoplasmic tyrosine kinase domain, which the latter is a key player in most cellular activities^[^^35^^]^. It has been reported that tumor progression can occur through regulating EMT in a variety of cancers. In the EMT process, epithelial cells acquire a mesenchymal phenotype, which has an important role in tumors. EMT can be induced by different growth factors, including EGF, FGF, PDGF and HGF, which can activate MET-signaling pathways. The MET-signaling pathway has been contributed to the development of several cancers. The HGF-MET-signaling pathway mediates the invasive growth of different malignancies such as head and neck squamous cell carcinoma, gastric cancer, colorectal cancer, and ESCC^[^^36^^]^. The HGF-dependent phosphor-rylation of the MET receptor is correlated with an enhanced potential of malignant cell migration and invasion^[^^37^^]^, introducing this pathway as a remarkable therapeutic target for the suppression of invasion and the spread of tumor cells. The HGF/MET axis, along with interaction with other tyrosine kinases, can activate multiple signaling cascades in the cells, including Wnt/ß-catenin, Janus kinase/STAT, RAS/MAPK, PI3K/AKT, and Src, which among others can take a highly important role in cancer development. These aforementioned phenomena can regulate several tumor behaviors such as invasion and metastasis. MET, as a cancer therapeutic target, functions as an oncogene. It can promote cell proliferation, invasion, and migration. MET kinase small-molecule inhibitors and antibodies targeting MET or HGF have revealed antitumor activities in preclinical models. Some of these agents are undergoing clinical development^[^^37^^]^. Therefore, MET-signaling inhibition can be potentially considered as a therapeutic modality for the treatment of different MET-activated human cancers. However, some limitations may exist for developing MET-pathway targeted agents. Most of the currently used MET inhibitors are multi-target instead of being MET-selective, and this may result in unwanted off-target toxicities^[^^38^^]^. Furthermore, using some MET inhibitors in patients may arise problems against ideal MET inhibition. Although different US Food and Drug Administration-approved agents (e.g., onartuzumab, tivantinib, and crizotinib) can block the HGF/MET pathway, specific agents against the MET/HGF signaling are not well-defined yet. MET overexpression has been reported in aggressive tumors such as gastric, renal, esophageal, and lung malignancies^[^^24^^,^^25^^]^. Therefore, MET kinase inhibitors are expected to provide a clinical beneﬁt in related patients^[^^39^^,^^40^^]^. Our results suggested that an effective dose of these compounds can be used as therapeutic agents. Except for D8, all heterocyclic compounds, which were used in this study, inhibited not only cell migration but also the autophosphorylation of the MET receptor in KYSE-30 cells. The D8 compound with a functional amino group (-NH2) had the lowest IC_50_ concentration (IC_50 _= 75.29 μM) among the other compounds of interest. Our results showed that D8 significantly (*p* = 0.05) inhibited cell growth in its IC_50_ concentration. It may imply that D8 could effectively reduce cell metabolism, division, and proliferation although its MET inhibitory effect was low. D1, D2, D5, D6, and D7 had the IC_50_ concentrations of 167 μM (*p* = 0.04), 556 μM (*p* = 0.03), 674 μM *p* = 0.006), 307 μM (*p* = 0.02), and 162 μM (*p* = 0.017), respectively. Based on the results, other compounds, including D1, D2, D5, D6, and D7, significantly decreased cell proliferation and growth, as well as the migration of KYSE-30. Furthermore, these compounds at an IC_50_ concentration reduced the autophosphorylation of Tyr1234 and Tyr1235 in the MET receptor, confirming their specificity for the MET receptor. Indeed, it is believed that compounds should have the highest efficiency with the lowest concentration in order to impose the minority of chemical changes and side effects *in vivo*. Since MET inhibition is important in drug selection, our compounds need to be structurally optimized to reach the MET receptor specificity. 

Based on the results, ESCC cell migration was significantly inhibited by D series of SMIs in the examined cell line. Considering that the level of MET phosphorylation was determined using the Western blot, and cell migration was also measured at different levels of MET phosphorylation states (by using our MET inhibitor compounds), we can combine the results and correlate the level of MET phosphorylation and migration in the examined cells. Our results showed that a weak intensity of the Western blot band, which describes a decreased MET autophosphorylation state, was correlated with the reduced distances of cell migration. In other words, MET phosphorylation leads to an increase in cellular migration in ESCC and lung cancer^[^^41^^]^. The obtained findings may confirm the role of MET in the migration of other malignancies. It has been shown that shikonin suppresses MET-mediated EMT and inhibits invasion and migration in human lung cancer cells^[^^41^^]^. MET mainly contributes to the invasion and migration of hepatocarcinoma cells, and its down-regulation is suggested as a therapeutic modality for the disease in experimental models^[^^42^^]^. Furthermore, the blocking of the HGF-MET pathway and angiogenesis by NK4 in malignant pleural mesothelioma significantly attenuates invasion and migration capacities^[^^43^^]^. Yang *et al.*^[^^44^^]^ have displayed that the crosstalk between FoxM1 and MET/AKT promotes EMT and plays a role in the invasiveness and migration of tongue squamous cell carcinoma. It has been frequently represented that microRNAs inhibit many types of cancer cell invasion and migration such as cervical^[^^45^^]^, breast^[^^46^^]^, non-small cell lung^[^^47^^]^, gastric^[^^48^^]^, ovarian, melanoma^[^^49^^]^, and osteosarcoma malignancies^[^^50^^]^ by targeting MET. It has also been revealed that the *in vitro* downregulation of MET phosphorylation at tyrosine residues by the silencing of 6-phosphogluconate dehydrogenase leads to the significant inhibition of cells. Consequently, 6-phosphogluconate dehydrogenase seems to be required for effective MET signaling and migration^[^^51^^]^. The oncogenic role of MET in ESCC and its role in the migration have been reported, as well^[^^52^^,^^53^^]^. Some HGF/MET inhibitors are currently used in the clinic, including crizotinib (suppresses the migration and invasion of the human NCI-H441 lung carcinoma cell), MK-2461 (a potent ATP-competitive inhibitor significantly reduces migration and tubulogenesis), and foretinib (EXEL-2880, which inhibits murine B16F10 melanoma cell migration and invasion). All these chemical compounds bind to the ATP-binding pocket of MET and block its activity, leading to the suppression of a variety of MET-dependent biological activities. In line with these reports, the results of the current study also revealed that the expression level of phosphorylated MET is significantly decreased by treatment with these novel compounds. Therefore, MET can be introduced as a potential therapeutic target for ESCC therapy. Although detailed investigations are required to identify MET function in ESCC. To the best of our knowledge, the presented compounds in this study (the D group) and their MET inhibitory potential have not been reported yet. 

In general, this study introduced some novel and potent MET inhibitor compounds with excellent kinase inhibition activity and effective potential for preventing the cell growth and migration. As far as we know, the present study is the first one that focused on the impact of phosphorylated MET on KYSE-30 cell migration ability, as well as the structure and function of novel compounds as new MET inhibitors for ESCC. The evidence may introduce such small inhibitors as potential therapeutic agents for repressing MET activity and inhibiting cancer cell proliferation and migration.
